# Insight on the larval habitat of Afrotropical *Culicoides* Latreille (Diptera: Ceratopogonidae) in the Niayes area of Senegal, West Africa

**DOI:** 10.1186/s13071-016-1749-1

**Published:** 2016-08-22

**Authors:** Mame T. Bakhoum, Assane G. Fall, Moussa Fall, Chiavaroli K. Bassene, Thierry Baldet, Momar T. Seck, Jérémy Bouyer, Claire Garros, Geoffrey Gimonneau

**Affiliations:** 1 Cirad, UMR CMAEE, Montpellier, France; 2 Institut Sénégalais de Recherches Agricoles, Laboratoire National de l’Elevage et de Recherches Vétérinaires, BP 2057, Dakar-Hann, Sénégal; 3 PATTEC coordination office, P. O. Box 3243, Addis Ababa, Ethiopia; 4 Cirad, UMR INTERTRYP, F-34398, Montpellier, France; 5 Present address: CIRDES, BP454, Bobo-Dioulasso, Burkina Faso

**Keywords:** *Culicoides*, Larval habitats, Flotation technique, Senegal, African horse sickness

## Abstract

**Background:**

Certain biting midges species of the genus *Culicoides* (Diptera: Ceratopogonidae) are vectors of virus to livestock worldwide. *Culicoides* larval ecology has remained overlooked because of difficulties to identify breeding sites, methodological constraints to collect samples and lack of morphological tools to identify field-collected individuals to the species level. After the 2007 unforeseen outbreaks of African horse sickness virus (AHSV) in Senegal (West Africa), there is a need to identify suitable and productive larval habitats in horse farms for the main *Culicoides* species to evaluate the implementation of vector control measures or preventive actions.

**Methods:**

We investigate twelve putative larval habitats (habitat types) of *Culicoides* inside and outside of three horse farms in the Niayes area of Senegal using a combination of flotation and emergence methods during four collection sessions.

**Results:**

Among the three studied horse farms, three habitat types were found positive for *Culicoides* larvae: pond edge, lake edge and puddle edge. A total of 1420 *Culicoides* individuals (519♂/901♀) belonging to ten species emerged from the substrate samples. *Culicoides oxystoma* (40 %), *C. similis* (25 %) and *C. nivosus* (24 %) were the most abundant species and emerged from the three habitat types while *C. kingi* (5 %) was only retrieved from lake edges and one male emerged from puddle edge. *Culicoides imicola* (1.7 %) was found in low numbers and retrieved only from pond and puddle edges.

**Conclusions:**

Larval habitats identified were not species-specific. All positive larval habitats were found outside the horse farms. This study provides original baseline information on larval habitats of *Culicoides* species in Senegal in an area endemic for AHSV, in particular for species of interest in animal health. These data will serve as a point of reference for future investigations on larval ecology and larval control measures.

## Background

Biting midge species of the genus *Culicoides* Latreille (Diptera: Ceratopogonidae) comprise around 1358 described species distributed worldwide [1]. Certain *Culicoides* species are known as the biological or putative vectors of viruses of domestic and wild ruminants as well as horses, such as the Schmallenberg virus (SBV), Akabane virus (AKAV), Bluetongue (BT) virus, epizootic haemorrhagic disease (EHD) virus and African horse sickness (AHS) virus [2, 3]. Vector control strategies for *Culicoides* spp. are needed. Indeed, vector control aims at reducing density of *Culicoides* populations at adult and larval stages, to limit host-vector contacts and hence decrease virus transmission [4, 5]. Among putative strategies, biological, chemical or environmental control of immature stages have been overlooked principally because oviposition behavior, breeding sites, larval habitats and factors regulating immature abundance are mostly unknown for *Culicoides* vector species. Baseline data on larval habitats of main vector species worldwide are needed to have a better understanding of their ecology and to provide new insights to the development of efficient vector control measures [6–9].

*Culicoides* spp. larval habitats are usually defined as humid rich and enriched in animal or vegetal organic matter, and may cover a wide range of natural and artificial substrates. Indeed, many larval habitats are described worldwide, including freshwater marshes and swamps, shallow margins of ponds, streams and rivers, bogs and peat lands, beaches, around leaking irrigation pipes and water troughs, tree holes and other natural cavities in rotting wood, waterlogged pastures, animal manure, rotting fallen fruits, highly alkaline or saline inland pools and animal dung [10–20].

Species diversity of *Culicoides* could be locally high [21–23], but it is admitted that only a limited number of species are able to transmit AHSV or BTV [2]. Consequently, larval ecology of only abundant vector species is described for livestock-related areas in some areas where the species occur [13, 23–25]. The main BTV vector in northern America, *C. sonorensis*is associated with edges of wastewater and polluted ponds on farms, but other aquatic sources (irrigation runoff in pasture, puddles, trough spillover) have also been found as high productive habitats [4, 23, 26]. In the Oriental and Australasian regions, *C. brevitarsis* larvae are mainly found associated with cattle dung [27, 28] unlike those of *C. oxystoma* which are found in aquatic and semi-aquatic habitats, such as paddy fields, stream edges, pond margins or estuary [28–30]. Larvae of the BTV vector species belonging to the subgenus *Avaritia* in the Palaearctic region (*C. obsoletus*, *C. scoticus*, *C. dewulfi*, *C. chiopterus* and *C. dewulfi*) are usually mentioned in the literature associated with cattle dung or cattle/sheep farm environment [13, 25, 31, 32], whereas *C. obsoletus* and *C. scoticus* could occupy a wide range of habitats inside and outside farm buildings [13, 32]. *Culicoides chiopterus* and *C. dewulfi* showed preferences for high soil moisture [33] and are referred as cattle dung breeders [34].

Certain species, e.g. *C. imicola*, have a wide range of larval habitats. Initially, larvae of *C. imicola* (affiliated to the subgenus *Avaritia*) are reported in permanent moist grassed margins of streams, furrows where grass is kept short by grazing animals in its southern historical distribution range (i.e. in South Africa) [17]. In northern Sardinia, an island in the Mediterranean, larval habitats of *C. imicola* are muddy habitats, not waterlogged, above unvegetated pond margins [24], which matches the first observations of *C. imicola* larval habitats in South Africa [18]. In Israel, Braverman et al. [11] described abundant larval populations in rich mixture of organic matter and water saturated soil, and concluded that this substrate was the favorable habitat of *C. imicola* (named by its synonym name *C. pallidipennis* in the article). Other descriptions of *C. imicola* larval habitats are detailed from Kenya [15], Nigeria [12] and Rhodesia (equivalent in territorial terms to modern Zimbabwe) [35], but suspected taxonomic uncertainty or misidentification [17] made the larval habitat descriptions doubtful for *C. imicola*. The anecdotal record of *C. imicola* by Nevill [36] breeding in cattle dung is also considered by Meiswinkel a misidentification [17]. Therefore, in the Afrotropical region, favorable larval habitats of *C. imicola*, the main vector species of AHSV and BTV, are only well described from South Africa. Apart from *C. imicola*, larval habitats of other Afrotropical species were investigated mostly in South Africa [14, 16, 18, 19], Zimbabwe [35] Nigeria [12] and Kenya [15]. These larval habitats can be grouped into four main types: (i) moist soil enriched greater or lesser in organic matter (decomposing plant matter, varying from intact material to humus, or of decomposed dung, such as is often found on irrigated pastures) with a great diversity of associated species [12, 15, 16, 18]; (ii) tree holes and other natural cavities in rotting wood, with often rare species such as *C. accraensis*, *C. inornatipennis*, *C. clarkei*, *C. confusus*, *C. eriodendroni*, *C. nigripennis*, C. *olyslageri* and *C. punctithorax* [16, 18]; (iii) dung pats of large herbivores such as African buffalo and cattle; and (iv) rotting fallen fruits of the sausage tree. The latter two larval habitat types are used respectively by *C. bolitinos* [16, 17] and *C. tuttifrutti* [16]. Overall, for the Afrotropical region, data on *Culicoides* larval habitats are scarce and mostly limited to southern Africa. Moreover, most of the studies have described favorable larval habitats in bovine environment areas [15–17, 35]. Despite the importance of AHSV in the African region, no larval habitats of *Culicoides* spp. are described in horse-surrounding ecosystems and no species are associated with horse dung.

Immature stages are localized at the substrate surface [37, 38]. Different techniques were used to investigate *Culicoides* spp. larval habitats and to determine the presence/absence and abundance of *Culicoides* larvae: sampling larvae from the substrate, emergence traps in the field and emergence pots. Larval collection methodology has been described and reviewed in [39] and sugar flotation provides the most effective results with low larval mortality [34]. Emergence methods are practiced by installing emergence traps directly in the field or by incubating the substrate samples in controlled environment with optimal conditions [12, 14, 15]. For larval ecology studies, a combination of both methods is necessary because no identification keys for immature stages exist for *Culicoides* spp. Recently, molecular identification of *Culicoides* larvae has been used through barcode sequences [28, 40]. This approach is powerful but is only applicable if the species diversity in a specific area has already been barcoded.

In Senegal, 53 species of *Culicoides* are recorded including species proven or suspected biological vectors of viruses of interest in animal health such as *C. imicola*, *C. bolitinos*, *C. kingi* and *C. oxystoma* [21, 22, 41, 42]. The country faced outbreaks of African horse sickness (AHS) in 2007 which caused the death of 1169 horses and considerable economic losses, estimated to 1.4 million euros [43, 44].

This study was designed to provide baseline information on larval habitats of Afrotropical *Culicoides* spp., in particular for species proven or suspected biological vectors of viruses in three horse farms in a known AHS-endemic region in Senegal. Identifying larval habitats in this area may serve as a point of reference for future investigations on larval ecology and larval control measures as already investigated in some regions [5, 7, 45].

## Methods

### Study sites

**Fig. 1:**
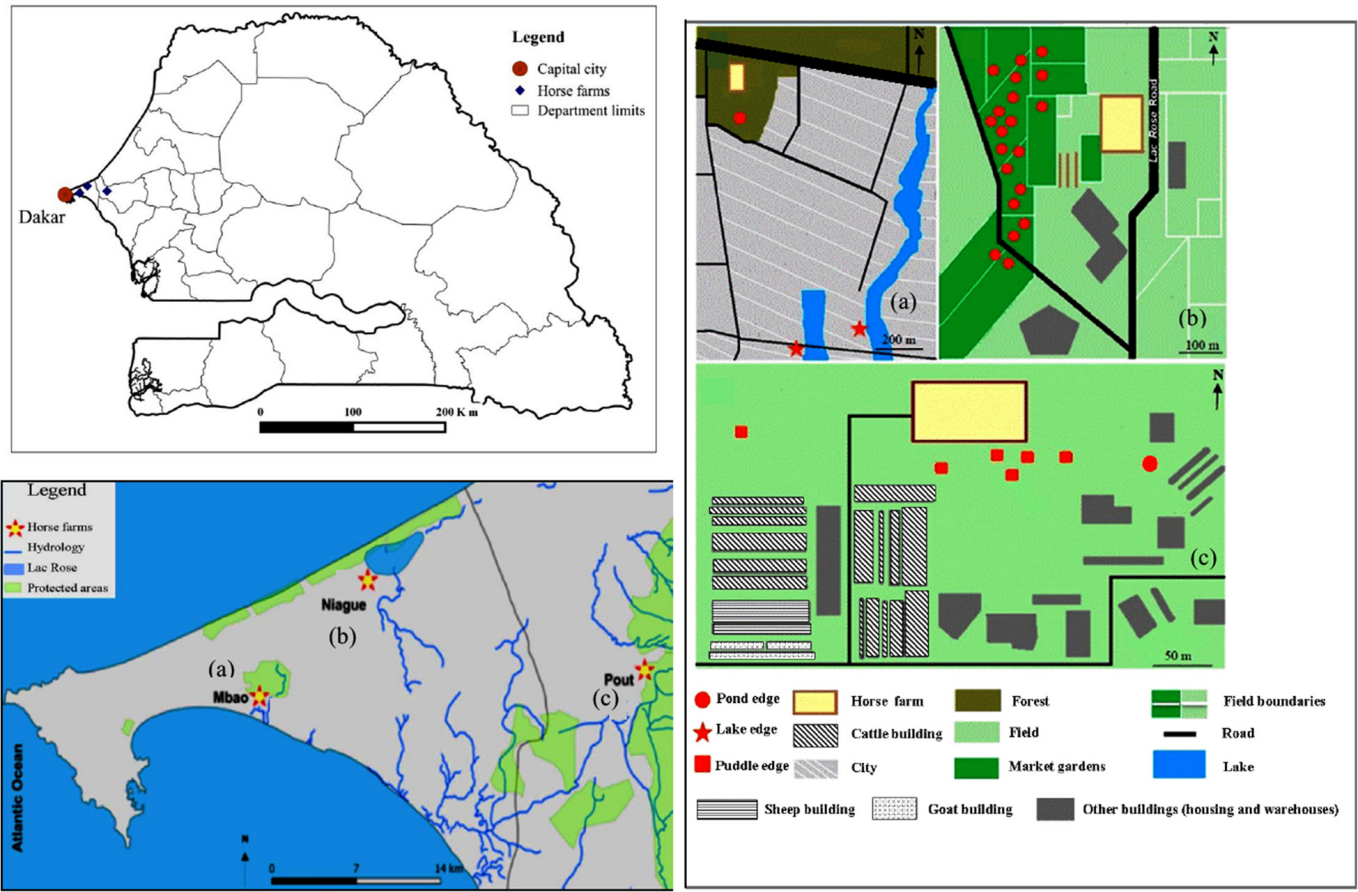
Map of the study horse farms in the Niayes area (Senegal). Sketch map of each horse farms are represented, **a** Mbao farm, **b** Niague farm and (**c**) Pout farm. The distance from *Lac Rose* to Niague farm is 1.2 km

The study was conducted in three horse farms affected by the 2007 AHS epizootic in the Niayes area in the vicinity of Dakar and Thiès, Senegal (Fig. 1) (see references [21, 42] for study region description): (**a**) Horse farm of Mbao (latitude: 14.7467, longitude: -17.3327) is a riding centre with 32 horses surrounded by a protected forested area. (**b**) Horse farm of Niague (latitude:14.8234, longitude: -17.2499) is a riding centre with 30 horses, 1 donkey and less than 10 cows or sheep, surrounded by a market-gardening area. (**c**) Horse farm of Pout (latitude: 14.7665, longitude: -17.0357) is a modern farm located in a rural environment. The latter site owns a wide range of animals in high numbers: 20 equids, 1700 sheep, 240 goats and 1600 cows or buffaloes. In each horse farm, dung and manure in animal buildings and boxes were removed every day and accumulated outside horse farms before being used for field spreading by local farmers. Previous entomological monitoring conducted in the three horse farms revealed high abundance and large diversity of *Culicoides* spp. [21, 22].

### Larval habitat classification, sample collection strategy and monitoring

**Fig. 2:**
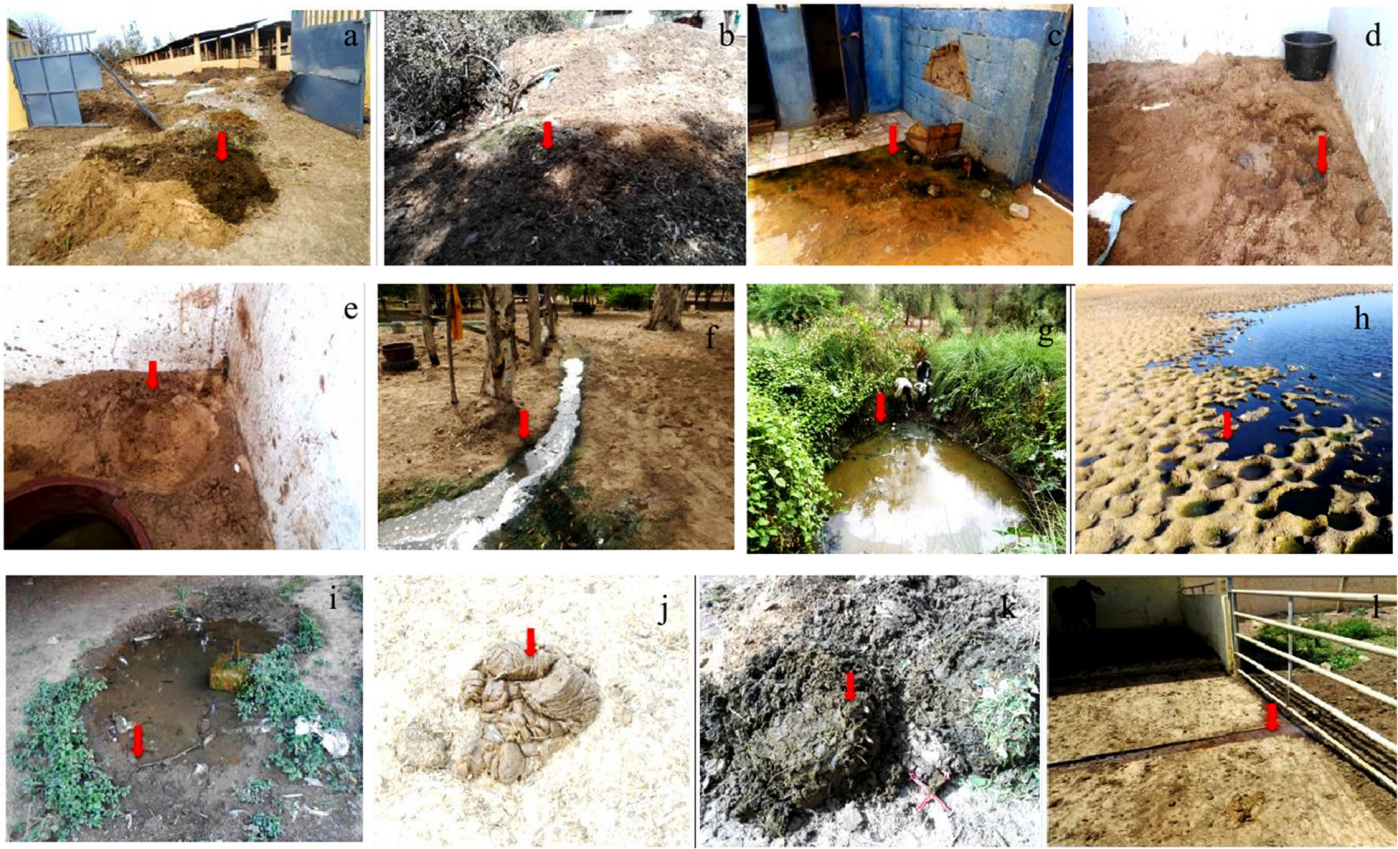
Pictures of the 12 *Culicoides* larval habitats investigated. The red *arrow* indicates where samples were collected. **a** Larval habitat 1 (outdoor fresh horse manure). The manure is wet, recently deposited on a heap. The manure was previously collected in horse boxes and is a mixture of sand and horse faeces. **b** Larval habitat 2 (outdoor old horse manure). Manure is a mixture of sand and horse faeces, partially dry and localized on a heap. **c** Larval habitat 3 (inside farm along protected fence). Samples were a mixture of manure and moist organic matter. **d** Larval habitat 4 (indoor litter soil). Solid and wet litter was collected inside horse boxes. Litter is a mix of sand and horse urine. **e** Larval habitat 5 (indoor humid soil). Solid and wet litter was collected inside horse boxes, under and around troughs. **f** Larval habitat 6 (water flow). Samples were collected at the edge of a water flow. Water resulted from washing horses and contained a lot of horse hairs, soap and organic matter (mainly faeces). Soil was sandy. **g** Larval habitat 7 (pond edge). Samples were collected at the interface between water and ground. Ponds were always located outside farms, with vegetation, and are used as water reserve for irrigation. Water is present all year along and soil was muddy and sandy. **h** Larval habitat 8 (brackish river and lake edges). Samples were collected at the interface between water and sandy soil. Water is highly polluted by riverine waste and presents a green/dark colour. **i** Larval habitat 9 (puddle edges). Samples were collected at the interface between water and ground. All the puddles sampled during our study results from water leak. Water was from a drilling and therefore does not contained chlorine. Soil was muddy. **j** Larval habitat 10 (fresh cattle dung). Samples were collected from dung deposited inside cattle boxes. **k** Larval habitat 11 (cattle dung heap). Samples were collected from a large cattle dung heap near the farm. Organic matter was humid. **l** Larval habitat 12 (liquid manure). Samples were collected from a flow channel of cattle liquid manure. It was a mix of urine and faeces

We have defined and described twelve putative larval habitats (habitat types) inside and outside horse farms with reference to the literature to match previous classifications [31] (Figs. 1 and 2). At each horse farm, all the twelve defined putative larval habitat types were investigated at several sampling sites if available (Table 1).

**Table 1:** Number of flotation for each of the twelve *Culicoides* larval habitats investigated according to horse farm and collection session

	Number of flotation/Horse farm/Collection session												
	16^th^ September			30^th^ September			15^th^ October			30^th^ October			
Larval habitat	A	B	C	A	B	C	A	B	C	A	B	C	Total
1. Outdoor fresh horse manure	3	2	3	2	–	–	–	–	–	–	–	–	10
2. Outdoor old horse manure	2	5	–	2	–	–	2	2	–	–	–	–	13
3. Inside farm along protected	–	2	–	–	–	–	–	–	–	–	–	–	2
4. Indoor litter soil	7	5	3	6	–	5	2	–	4	–	–	4	36
5. Indoor humid soil	6	2	2	4	–	2	–	–	–	–	–	–	16
6. Water flow	2	–	–	–	–	–	–	–	–	–	–	–	2
7. Pond edge	1	6	–	1	–	–	1	9	–	1	5	–	24
8. Lake edge	–	–	–	1	–	–	2	–	–	2	1	–	6
9. Puddles edge	4	–	2	6	–	5	–	–	4	–	–	3	24
10. Fresh cattle dung	–	2	–	–	–	4	–	2	4	–	–	2	14
11. Cattle dung heap	–	–	–	–	–	3	–	–	2	–	–	2	7
12. Liquid manure	–	–	–	–	–	3	–	–	2	–	–	2	7
Total	25	24	10	22	–	22	7	13	16	3	6	13	161

*Abbreviations*: *A* horse farm of Mbao, *B* horse farm of Niague, *C* horse farm of Pout

Samples were collected in September-October 2014 during four collection sessions (16th September, 30th September, 15th October and 30th October), corresponding to the end of the rainy season. For each available defined habitat type (Fig. 2, Table 1), one substrate sample of approximately 650 cm^3^ was collected in the upper layer of soil surface (0–5 cm) with a trowel, filtered with a fine mesh sieve of 0.8 mm diameter and then investigated for midge larvae in the field using a direct flotation technique in saturated sugar solution (850 g/l). Presence/absence of midge larvae was used as a proxy for positive larval habitat. If no midge larvae were observed in the first flotation sample, a second replicate was collected if possible. For each positive flotation sample, up to three samples of approximately 125 cm^3^ were collected from the 0–5 cm of soil using a trowel and placed in 200 ml plastic pots covered with a net before being transported to the laboratory (insectarium) to monitor adult emergence. Emerging pots were maintained for 21 days at a temperature of 25 ± 1 °C, relative humidity of 80 ± 10 % and a light:dark photoperiod of 12:12 h to allow the retrieval of emerging adult *Culicoides*. The surface of the substrate was sprayed every two days with demineralized water to prevent desiccation. Each day, emerging adults were collected using a mouth-operated aspirator and then preserved in 90° ethanol. *Culicoides* species identifications were done on emerged adults using a stereomicroscope (10–40×) and reference identification keys [46–48].

## Results

For the three horse farms and the four collection sessions, a total of 161 flotation samples were collected (Table 1). Of these, 45 samples (28 %) were positive for *Culicoides* spp. larvae, which resulted in 135 emergence pots.

Among the 12 putative larval habitats, three larval habitats were found positive for *Culicoides* larvae using sugar flotation collection method: (**g**) pond edge, (**h**) lake edge and (**i**) puddle edges (Fig. 2). No larvae were retrieved of samples from putative larval habitats associated with horse faeces; from a mixture of organic matter and water and from putative larval habitats associated with cattle dung (Fig. 2). All of the positive sampling sites for these three larval habitats were localized outside horse farms (Fig. 1).

**Fig. 3:**
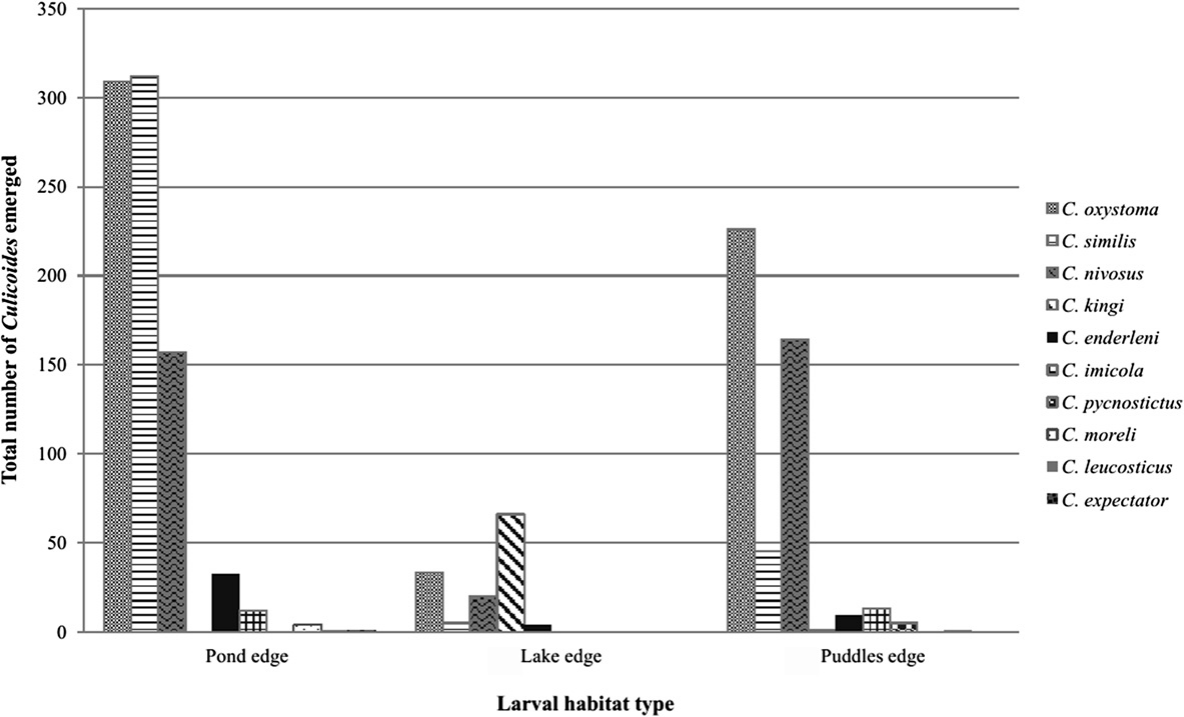
Number of emerged individuals per species for each larval habitat type

**Fig. 4:**
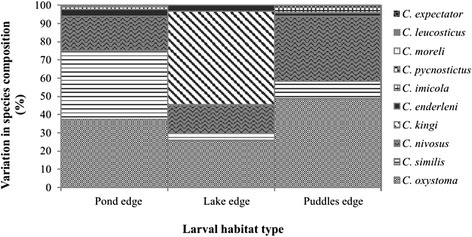
Species composition for each larval habitat type

**Fig. 5:**
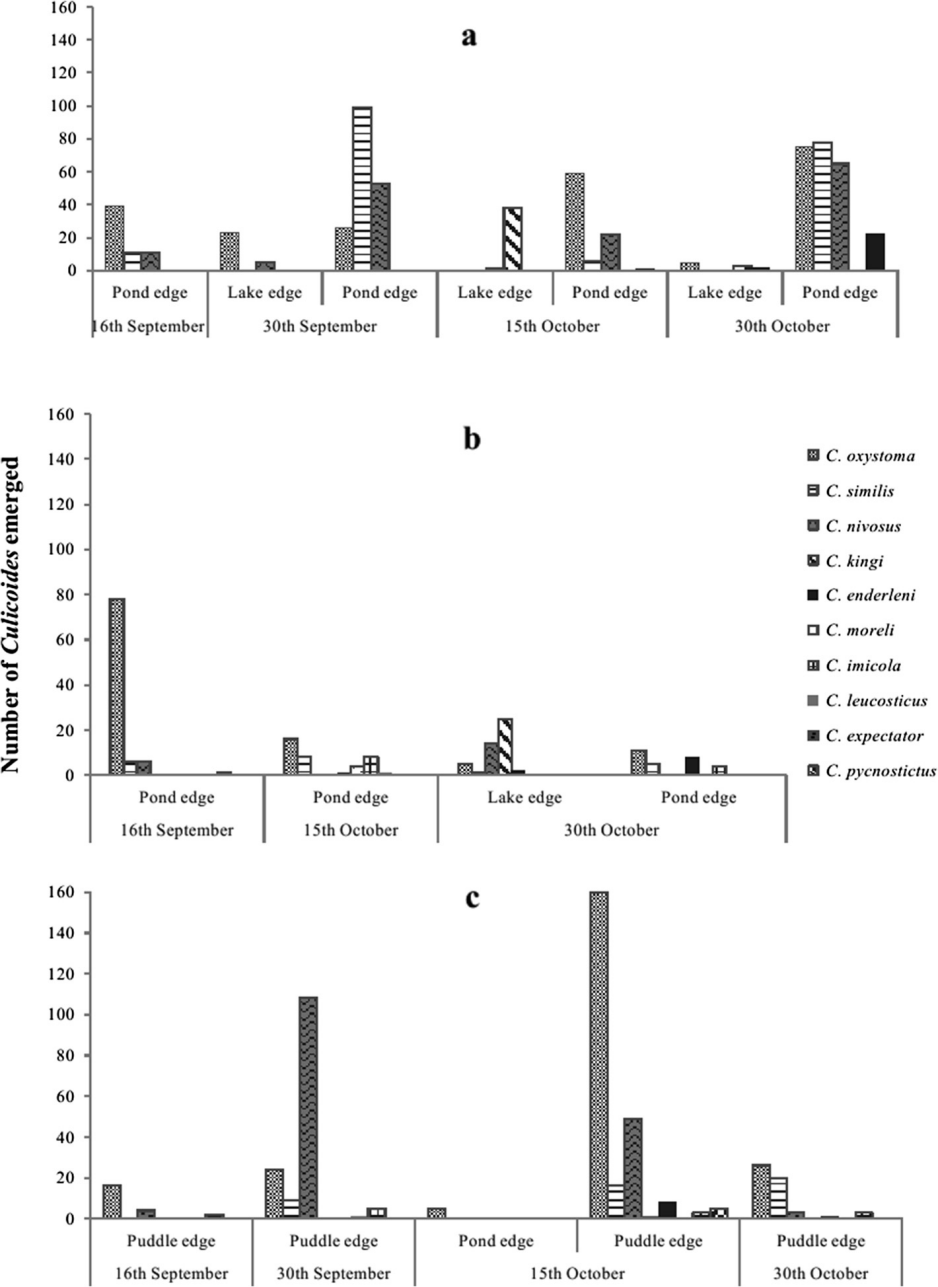
Emergence dynamics at the positive larval habitats at each horse farm according to collection session. **a** Mbao farm, **b** Niague farm and **c** Pout farm

A total of 1420 adult *Culicoides* (519 ♂/901 ♀) belonging to 10 species emerged from the 135 substrate samples in the laboratory (Tables 2 and 3; Figs. 3, 4 and 5) together with other dipteran species belonging to the families Ceratopogonidae (genus *Forcipomyia*) and Psychodidae (data not shown).

**Table 2:** Mean (minimum-maximum) number of *Culicoides* individuals emerged from substrate samples per species, site and larval habitat type

Species/Site	Pond edge			Lake edge			Puddle edge			Total number
	A^a^	B^b^	C^a^	A^c^	B^a^	C	A	B	C^d^	
*C. oxystoma*	49.8 (26–75)	26.3 (3–78)	1.3	10.8 (5–23)	1.3	–	–	–	56.5 (16–160)	568
*C. similis*	48.5 (6–99)	29.5 (3–54)	–	–	1.3	–	–	–	11.3 (9–20)	362
*C. nivosus*	37.8 (11–65)	1.5	–	1.5 (1–5)	3.5	–	–	–	41 (3–108)	341
*C. kingi*	–	–	–	10.3 (3–38)	6.3	–	–	–	0.3	67
*C. enderleini*	6.5 (1–22)	2.3 (1–8)	–	0.5	0.5	–	–	–	2.3 (1–8)	45
*C. imicola*	–	3 (1–7)	–	–	–	–	–	–	3.3 (2–5)	25
*C. pycnostictus*	–	–	–	–	–	–	–	–	1.3	5
*C. moreli*	–	1	–	–	–	–	–	–	–	4
*C. leucosticus*	–	0.3	–	–	–	–	–	–	0.3	2
*C. expectator*	–	0.3	–	–	–	–	–	–	–	1

*Abbreviations*: *A* horse farm of Mbao; *B* horse farm of Niague, *C* horse farm of Pout

^a^One sampling site sampled

^b^Four to six sampling sites sampled

^c^One to two sampling sites sampled

^d^Four to five sampling sites sampled

**Table 3:** Sex ratio (SR) of *Culicoides* individuals emerged from substrate samples per species, horse farm and larval habitat type

	Pond edge			Lake edge			Puddle edge			
Species/Sites	A	B	C	A	B	C	A	B	C	Total
*C. oxystoma*	63 ♂/136 ♀	38 ♂/67 ♀	2 ♂/3 ♀	9 ♂/19 ♀	5 ♀	–	–	–	100 ♂/126 ♀	212 ♂/356 ♀
*C. similis*	75 ♂/119 ♀	52 ♂/66 ♀	–	–	5♀	–	–	–	17 ♂/28 ♀	144 ♂/218 ♀
*C. nivosus*	36 ♂/115 ♀	4 ♂/2 ♀	–	1 ♂/5 ♀	5 ♂/9 ♀	–	–	–	72 ♂/92 ♀	118 ♂/223 ♀
*C. kingi*	–	–	–	21 ♂/20 ♀	4 ♂/21 ♀	–	–	–	1 ♂	26 ♂/41 ♀
*C. enderleini*	3 ♂/20 ♀	2 ♂/7 ♀	–	2 ♀	1 ♂ /1 ♀	–	–	–	2 ♂/7 ♀	8 ♂/37 ♀
*C. imicola*	–	2 ♂/10 ♀	–	–	–	–	–	–	4 ♂/9 ♀	6 ♂/19 ♀
*C. pycnostictus*	–	–	–	–	–	–	–	–	2 ♂/3 ♀	2 ♂/ 3 ♀
*C. moreli*	–	4 ♀	–	–	–	–	–	–	–	4 ♀
*C. leucosticus*	–	1 ♂	–	–	–	–	–	–	1 ♂	2 ♂
*C. expectator*	–	1 ♂	–	–	–	–	–	–	–	1 ♂
Total	177 ♂/390 ♀	100 ♂/156 ♀	2 ♂/3 ♀	31 ♂/46 ♀	10 ♂/41 ♀	–	–	–	199 ♂/265 ♀	519 ♂/901 ♀ (SR = 0.58)

*Abbreviations*: *A* horse farm of Mbao; *B* horse farm of Niague; *C* horse farm of Pout

For the three positive larval habitats, 90 % of the emerged specimens belong to three species: *C. oxystoma* (*n* = 568), *C. similis* (*n* = 362) and *C. nivosus* (*n* = 341) (Table 2). Less than 2 % of the emerged *Culicoides* were identified as *C. imicola* (25 individuals) in horse farms of Niague and Pout from pond edge and puddle edge, respectively (Tables 2 and 3). *Culicoides kingi* emerged almost exclusively in horse farms of Mbao and Niague from lake edge; only a single individual emerged from puddle edge in horse farm of Pout. The density of emerged species varied according to larval habitats (Figs. 3 and 4).

The overall sex ratio was unbalanced towards females (SR = 0.58), which was also observed for the two most abundant species (*C. oxystoma* and *C. similis*) and all of the positive larval habitats (Table 3). At horse farms of Mbao and Niague, the most productive larval habitats were pond edges (88 and 83 % of the emerging adults, respectively) (Fig. 3, Table 2). At horse farm of Pout, 99 % of the emerging adults (both sexes) were obtained from puddle edge (Table 2). The highest number (*n* = 644) of emerging individuals was found at horse farm of Mbao, followed by horse farm of Pout (*n* = 469) and horse farm of Niague (*n* = 307) (Table 3). The study of emergence dynamics showed marked variations between sites, larval habitats and species (Fig. 5), probably due to the different environmental and meteorological conditions.

## Discussion

Larval habitats of *Culicoides* vector species are investigated in many areas in the world where the species occur. In the Afrotropical region where AHSV is endemic, larval habitats of *Culicoides* are almost unknown or require an update except for the southern part of the continent where *Culicoides* larval habitats are much investigated [14, 16, 19].

To our knowledge, this study is the first to identify larval habitats of several *Culicoides* species, including the species potentially involved in AHSV transmission, in the Niayes area, Senegal, West Africa, in horse-related ecosystems. Positive larval habitats were recorded only outside horse farms. No species-specific habitats were identified although *C. kingi* was particularly abundant in lake edge habitats in horse farms of Mbao and Niague; just one individual emerged from puddle edge in horse farm of Pout. Since the last outbreak of AHSV in 2007 in Senegal, several studies have been conducted in the same horse farms in the Niayes area to characterize *Culicoides* species diversity [21, 41, 42], trophic behaviour [42, 49–51] and population dynamics [21, 22] at the adult stage. These surveys conducted on adult populations in these three horse farms using suction black-light traps revealed a high species diversity with at least 18 species collected [21, 42], whereas 10 species emerged from the collected substrates in our study. Of these 10 species, eight are proven or suspected biological vectors of viruses of interest in animal health: *Culicoides imicola* major vector of AHSV in Afrotropical region [52–55], *C. oxystoma* [56, 57], *C. kingi*, *C. enderleini*, *C. nivosus*, *C. leucostictus*, *C. pycnostictus* and *C. exspectator* [55]. *Culicoides kingi* is involved in the transmission of *Onchocerca gutturosa*, a parasite of cattle [58].

It is known that the abundance of *Culicoides* larvae collected or emerged from substrates is lower than that of *Culicoides* adults collected using suction black-light traps. Thus, abundance of *C. sonorensis* adults collected in two dairy farms in Northern California using suction black-light traps was weakly correlated with that of the larvae [23]. Auriault et al. [59] followed during three years *C. grahamii* populations at both adult and larval stages in Gabon. These authors did not identify the larval habitats of this species using emergence traps in an environment of debris and decomposing banana trunks while *C. grahamii* adult populations were abundant and caused a nuisance to humans [59].

Interestingly, only semi-aquatic freshwater and saltwater habitats were positive for *Culicoides* larvae in our study; these were similar to some described larval habitats in South Africa [14, 18] and Kenya [15]. Other larval habitats mentioned in the Afrotropical literature such as the dung pats of large herbivores [16, 17] were found negative for *Culicoides* larvae in our study. Indeed, no larvae were found in horse and cattle dungs investigated in our study and all larval habitats identified were located outside the horse farms, in the immediate surroundings, and all represented permanent semi-aquatic habitats.

The absence of positive larval habitats inside horse farms may be due to the daily mechanical disturbance of horse litter. This may affect and limit the attraction of gravid females to these putative breeding sites and oviposition site choice. Indeed, mechanical disturbance may affect larval development of biting midges [60]. Moreover, insecticides are regularly sprayed in some horse boxes against arthropod vectors which could affect adult *Culicoides* oviposition and survival. Several studies have examined insecticides against *Culicoides* spp. and depending on the specific product; their efficacy to reduce attack rates and the survival of *Culicoides* spp. was more or less satisfying [60–62].

In our study, the overall sex ratio was unbalanced towards females, which is also observed for other studies [14, 32]. The numbers of emerged individuals and the composition of *Culicoides* spp. varied according to larval habitats and horse farms but also collection sessions (16th September, 30th September, 15th October and 30th October). *Culicoides kingi* and *C. imicola*, two important species of veterinary importance, emerged in very low numbers from field-collected substrate samples whereas adult specimens were abundantly collected at the same period in the same sites using suction black-light traps [21, 22]. The same was observed for *C. imicola* in South Africa by [14, 18]. Different hypothesis could explain these discrepancies. The developmental success and emergence rates might have been impacted in emerging pots in the laboratory. Even though larvae were reared in substrates from their natural habitats, the transfer of samples into the pot, and then from the field to the lab, changes the environmental conditions and may have induced mortality. Moreover, favourable larval habitats of *C. imicola* may have been probably poorly sampled or not sampled during the field investigations.

*Culicoides kingi* and *C. oxystoma* were the dominant species found around the brackish lake edges in Mbao and Niague. Further analyses of the substrate physico-chemical properties may provide a better understanding of specific environmental requirements for these species. *Culicoides oxystoma* was found in all positive larval habitats (pond edge, lake edge and puddle edge). Larvae of this species were also found in several aquatic and semi-aquatic habitats in Japan and India, such as paddy fields, stream edges and pond margins [28–30]. *Culicoides oxystoma* has a wide range of larval habitats such as the case of *C. imicola* in several areas where this species is present [14, 16, 18, 24].

## Conclusions

The combination of flotation and emergence methods was used to investigate 12 putative larval habitats; of these, three larval habitats were found outside the horse farms, in the immediate surroundings and represented permanent semi-aquatic habitats. No larvae were retrieved from larval habitat types associated with dung (horse dungs, fresh and heap cattle dungs). Although preliminary, these baseline results are very important to further insights into the larval habitats of *Culicoides* spp. in the Niayes area, an AHS endemic region in Senegal, before conducting further studies.

## Abbreviations

BTV: Bluetongue virus
AHSV: African horse sickness virus
AHS: African horse sickness
